# Physical activity monitoring in obese people in the real life environment

**DOI:** 10.1186/1743-0003-6-47

**Published:** 2009-12-30

**Authors:** Maria Grazia Benedetti, Annalisa Di Gioia, Linda Conti, Lisa Berti, Linda Degli Esposti, Giulietta Tarrini, Nazario Melchionda, Sandro Giannini

**Affiliations:** 1 Movement Analysis Laboratory, Istituto Ortopedico Rizzoli, University of Bologna, Italy; 2 Unit of Metabolic Diseases, Clinical Medicine Department, University of Bologna, Italy

## Abstract

**Background:**

Obesity is a major problem especially in western countries and several studies underline the importance of physical activity to enhance diet. Currently there is increasing interest in instruments for monitoring daily physical activity. The purpose of this pilot study was to appraise the qualitative and quantitative differences in physical activities and gait analysis parameters in control and obese subjects by means of an innovative tool for the monitoring of physical activity.

**Methods:**

Twenty-six obese patients, 16 women and 10 men, aged 22 to 69 years with Body Mass Index (BMI) between 30 and 51.4 kg/m^2^, were compared with 15 control subjects, 4 men and 11 women, aged 24 to 69 with BMI between 18 and 25 kg/m^2 ^during daily physical activities. The IDEEA device (Minisun, Fresno, CA), based on a wearable system of biaxial accelerometers and able to continuously record the physical activities and energy expenditure of a subject in time was used. Time spent in different physical activities such as standing, sitting, walking, lying, reclining, stepping, energy expenditure and gait parameters (velocity, stance duration, etc) were measured during a 24-hours period.

**Results:**

A trend toward a reduced number of steps was present, associated to reduced speed, reduced cadence and reduced rate of single and double limb support (SLS/DLS). Moreover, obese people spent significant less time stepping, less time lying and more time in a sitting or reclined position during the night. The energy expenditure during a 24-hours period was higher in the obese compared to controls.

**Conclusions:**

The study provided objective parameters to differentiate the daily motor activity of obese subjects with respect to controls, even a larger population is required to confirm these findings. The device used can be of support in programming educational activities for life style modification in obese people as well as for monitoring the results of various kinds of intervention in these patients concerning weight and physical performance.

## Background

A large amount of data are available on the relationship between physical activity and obesity [1-4]. In particular the National Institute of Health, in the "Clinical Guidelines on the Identification, Evaluation and Treatment of Overweight and Obesity in Adults" [1] highlights the importance of physical activity in weight loss. Physical activity increases energy expenditure, inhibits the sense of hunger, helps in maintaining optimal weight and reduces the risk of cardio-vascular disease. Guidelines also highlight the importance of the association of physical activity with diet, because it allows greater weight loss and greater BMI reduction than diet alone. Finally, the improvement of cardio-respiratory fitness and the reduction of cardio-vascular risk brought about by physical activity, independently of weight loss is emphasized.

In 2006 the Cochrane Library published a review [4] that underlined, through the meta-analysis of 43 studies, that physical exercise, especially high-intensity exercise, associated with diet is more effective than diet alone not only in determining weight loss, but also in decreasing the risk of cardiovascular disease.

Thus it is easy to understand the increasing interest in the development of tools able to monitor physical activity and reflect the complexity of human activities in daily life conditions.

Nowadays, the most commonly used methods for the measurement of daily physical activity are questionnaires, like the Physical Activity Scale for Elderly (PASE) [5], the NUGENOB study [6], the Baecke Questionnaire [7] and the International Physical Activity Questionnaire (IPAQ) [8]. Several studies have confirmed the validity and reliability of such questionnaires, by showing that their scores are related to the real daily physical activity of the subject. However, other authors underline the limits of such questionnaires, because they are strictly linked to the subjectivity of the evaluation - patients often tend to over-estimate the intensity or the duration of their own activity [9] - and to the lack of international standards to define the concept of "physical activity" and its intensity [10].

Moreover, technology currently offers numerous tools to measure objectively the level of physical activity (type, time, intensity, ...) performed by the subject during daily life or the daily energy expenditure, or even to associate these two measurements. Among the most widely used instruments are of course accelerometers (mono-, bi- or tri-axial), pedometers, gyroscopes, and heart rate monitors [11-13].

The aim of this pilot study was to compare the daily physical activity of a group of obese subjects with the daily physical activity of a group of control subjects. For this purpose an advanced wearable device, IDEEA, was used. It provides information about motor activities with regards to type and duration, gait parameters and energy expenditure and has been previously reported to be very accurate in adults [14-21].

## Methods

### Subjects

Twenty-six volunteer obese patients were enrolled on a voluntary basis from a wider group of subjects treated at the Department of Metabolism Disease of the University of Bologna (Italy). Sixteen were women and 10 men, aged 22 to 69 with a Body Mass Index (BMI) of between 30 and 51.4 kg/m^2^. Exclusion criteria were severe cardiovascular diseases and neurological impairment. The occupation of the obese subjects was as follows: employees n.11, nurses n.2, housewives 2, sales agent n.2, student n.2, retired n.2, truck driver n.1, worker n.1, bartender n.1, sales representative n.1, warehouse worker n.1.

Fifteen control subjects, 4 men and 11 women, aged 24 to 69 with a BMI of between 18 and 25 kg/m^2 ^were recruited (Table 1). Control subjects were on a voluntary base were enrolled among acquaintances and staff of the participating institutions, based on their BMI, on the absence of any known pathology and on their normal life style (no professional sports). Their current occupation was as follows: students n.5, physicians n.4, engineers n.3, physical therapist n.1, employee n.1, assistant researcher n.1.

**Table 1:** Characteristics of subjects

	Age Range (yrs)	Mean age (yrs)	Sex	Height Range (cm)	Mean height (cm)	Weight Range (kg)	Mean weight (kg)	BMI Range (kg/m^2^)	Mean BMI (kg/m^2^)
Norm (n.15)	24-69	45	11 F 4 M	155-186	168.8	47-97	61.8	18-25	21.33

Obese (n.26)	22-69	47,5	16 F 10 M	150-181	164.6	78-136	97.15	30-51.44	35.77

All subjects included were asked to spend the day performing all the usual activities both relative to their occupation and normal activities of daily life.

Informed consent was signed by all the subjects and the study was approved by the Ethical Committee of the project leader's Institute.

### Monitoring physical activity

IDEEA (Minisun, Fresno, CA) was used to measure energy expenditure and monitor physical activity. It is an integrated portable system, composed of a recording device (IDEEA data recorder, weighing 200 g, with a 33 MHz, 32 bit microprocessor) that allows the acquisition, processing and compression of data in real time - connected to 5 small DC coupled accelerometers (16 × 14 × 6 mm), that measure the angle between body segments and their acceleration on two planes, with a data acquisition frequency of 32 Hz (0,3 measures/s). For communication between the PC and the IDEEA recorder, the software allows data to be downloaded on the PC, which can be processed and visualized through the Act View program.

IDEEA provides information about different physical activities (standing, sitting, walking, lying, reclining, stepping, etc), walking speed, and energy expenditure produced during activities (EE - measured in kcal/min or kJ/min or Watts). The ability of IDEEA to appraise the energy expenditure under daily life conditions depends on its capacity to identify type and intensity of the activity developed and to connect these parameters with a series of equations for measuring energy expenditure for each activity.

The tool also provides gait analysis data, by identifying step and stride length, velocity, cadence, stance, swing and double support duration and visualizing the results through graphs and charts. Other parameters provided by the instruments are the "pulling acceleration", defined as the maximum linear acceleration of the foot during the initial swing phase, the "swing power", defined as the maximum deceleration during the mid and terminal phases of swing, and the "ground impact", defined as the maximum deceleration in a vertical direction during the loading acceptance [22].

Both control and obese subjects wore the IDEEA: sensors were positioned under the plantar arch of each foot, two on the anterior part of each thigh and one on the sternum, all fixed to the skin by a hypoallergenic adhesive tape. The tool was then connected to the PC, and the subject's anthropometric data were entered. At the end of this phase, the subject sat with the trunk in a vertical position, and feet and knees parallel to the floor (angles formed by hip, knee and ankle were 90°). In this position the device was calibrated according to the manufacturer's instructions [13]. After that the tool was disconnected from the computer and the patients were asked to lead their usual life for the following 24 hours and return the next day to download and process data.

### Statistic analysis

Statistical analysis was performed using the Mann Whitney test (p < 0.05) for non-parametric data with different variance.

## Results

### Motor activity

Considering all the physical activities, the obese subjects covered 4.63 ± 2.22 km (range 0,69-9,54 km) within the 24 hours, while control subjects covered 7.00 ± 3.81 km (range 2.93-14.80 km) (p = 0.09) (Table 2). Concerning the time spent in different physical activities, both in terms of percentage and absolute time, no significant difference between the two groups, either for walking or standing was found.

**Table 2:** Time spent in physical activities

	(%)				(min)			Mann Whitney	
	
		Min.	Mean ± SD	Max.	Min.	Mean ± SD	Max	%	min
Lie	Norm (n.26)	5.50	23.24 ± 9.24	35.90	74.30	327.10 ± 127.79	506.30	NS	NS
			
	Obese (n.15)	0.09	18.43 ± 12.24	44.70	1.20	263.31 ± 174.06	637.40		

Recline	Norm (n.26)	0.96	8.51 ± 6.70	26.67	13.80	119.94 ± 95.08	384.30	NS	NS
			
	Obese (n.15)	0.46	11.93 ± 8.79	31.83	6.70	184.86 ± 135.86	452.50		

Sit	Norm (n.26)	24.37	39.88 ± 10.33	60.76	355.40	562.30 ± 144.55	820.50	NS	NS
			
	Obese (n.15)	12.29	43.45 ± 12.78	71.76	172.20	621.20 ± 181.62	1033.8		

Stand	Norm (n.26)	15.13	21.53 ± 4.89	32.03	210.80	305.04 ± 75.48	461.3	NS	NS
			
	Obese (n.15)	11.09	20.58 ± 7.73	39.23	158.60	294.32 ± 110.99	569.9		

Walking	Norm (n.26)	2.49	5.61 ± 2.83	11.75	34.60	79.74 ± 41.64	171.40	NS	NS
			
	Obese (n.15)	0.9	4.55 ± 1.77	7.79	12.60	65.15 ± 25.31	109.20		

Step	Norm (n.26)	0.15	0.54 ± 0.52	2.26	1.18	5.54 ± 4.72	12.80	p = 0,05	p = 0,03
			
	Obese (n.15)	0.04	0.29 ± 0.35	1.37	0.56	2.94 ± 2.39	11.00		

Obese subjects spent 0.29 ± 0.35% of the time in stepping (range 0.04-1.37%, corresponding to 2.94 ± 2.39 min (range 0.56-11.0 min), while control subjects spent 0.54 ± 0.52% (range 0.15-2.26%) of the time, corresponding to 5.54 ± 4.72 min (range 1.18-12.80 min) (p = 0, 05, p = 0,03).

Even data about lying, sitting and reclining postures were not significantly different, when we considered only the night time hours (from 11:00 pm to 7:00 am), differences among the two groups were found (Table 3). The supine position was preferred by control subjects, with a percentage of 57.71 ± 22.74% (range 14.84-93.49%) against the 41.53 ± 27.38% (range 2.5-93.88%) of obese patients (p = 0.06). However no significant differences in the percentages of night time spent sitting or reclining between the two groups was found.

**Table 3:** Percentage of time spent lie or recline during the night *

	Lie (%)			Reclined (%)			Sitting (%)		
	
	Min	Mean ± SD	Max	Min	Mean ± SD	Max	Min	Mean ± SD	Max
Norm (n.15)	14.84	57.71 ± 22.74	93.49	0.07	15.16 ± 14.06	41.18	1.78	22.96 ± 14.52	49.07

Obese (n.26)	2.5	41.53 ± 27.38	93.88	0.16	20.72 ± 20.84	83.26	1.77	31.50 ± 22.85	94.01

Mann Whitney	p = 0.05			NS			NS		

* from 11.00 pm to 7.00 am

### Time-distance parameters of gait

The number of steps walked during the 24 hours was not significantly different in the two groups even a trend toward a reduced number of steps was evident in the obese group (5870.15 ± 2693.95 vs 7859.27 ± 4596.0 for the controls) (Table 4). Data about the total duration of the walk were not significant but even in this case a trend toward reduction in the obese subjects is evident 69.15 ± 25.80 min vs 84.13 ± 42.84 min for controls) - likewise for the distances covered walking (4.73 ± 2.53 km vs 5.90 ± 3.47 km for controls).

**Table 4:** Time distance parameters

		Mean ± SD	Min	Max	Mann Whitney
Steps	Norm (n.15)	7859,27 ± 4596.0	2177	17685	NS
		
	Obese (n.26)	5870.15 ± 2693.95	1102	11563	

Duration (min)	Norm (n.15)	84.13 ± 42.84	35	180	NS
		
	Obese (n.26)	69.15 ± 25.80	15	112	

Distance (km)	Norm (n.15)	5.90 ± 3.47	0.39	12	NS
		
	Obese (n.26)	4.73 ± 2.53	0.71	12.9	

Stride Length (m)	Norm (n.15)	1.81 ± 0.53	1.2	3.0	NS
		
	Obese (n.26)	1.64 ± 0.75	1.2	5.2	

Step Length (m)	Norm (n.15)	0.90 ± 0.26	0.62	1.48	NS
		
	Obese (n.26)	0.82 ± 0,37	0.58	2.59	

Swing (ms)	Norm (n.15)	433.54 ± 104.73	375.00	798.5	NS
		
	Obese (n.26)	409.47 ± 71.57	346.80	720.9	

Step (ms)	Norm (n.15)	553.22 ± 57.97	493.70	739.0	NS
		
	Obese (n.26)	590.95 ± 76.65	517.5	929.6	

Cycle duration (s)	Norm (n.15)	1.12 ± 0.09	1.00	1.36	p = 0.036
		
	Obese (n.26)	1.18 ± 0.07	1.05	1.32	

Cadence (steps/min)	Norm (n.15)	104.18 ± 10.06	82.0	119.40	p = 0.05
		
	Obese (n.26)	93.11 ± 20.11	8.10	112.00	

Speed (m/min)	Norm (n.15)	79.32 ± 19.44	61.20	141.6	p = 0.04
		
	Obese (n.26)	66.04 ± 19.83	44.4	150.5	

Single Support (ms)	Norm (n.15)	433.54 ± 104.73	375.0	798.5	NS
		
	Obese (n.26)	409.47 ± 71.57	346.80	720.9	

Double Support (ms)	Norm (n.15)	156.60 ± 68.63	96.10	393.4	NS
		
	Obese (n.26)	187.68 ± 57.9	131.20	420	

SLS/DLS (%)	Norm (n.15)	315.74 ± 50.16	211	441.30	p < 0.0005
		
	Obese (n.26)	241.76 ± 37.08	172.2	304.20	

Pulling Accel (G)	Norm (n.15)	0.62 ± 0.39	0.21	1.86	NS
		
	Obese (n.26)	0.63 ± 0.17	0.35	0.92	

Swing Power (G)	Norm (n.15)	0.95 ± 0.18	0.73	1.32	NS
		
	Obese (n.26)	0.90 ± 0.20	0.54	1.49	

Ground Impact (G)	Norm (n.15)	1.31 ± 0.17	0.92	1.53	NS
		
	Obese (n.26)	1.21 ± 0.34	0.05	1.82	

Pre swing angle (deg)	Norm (n.15)	50.14 ± 15.31	23.0	72.5	p = 0.03
		
	Obese (n.26)	37.54 ± 18.02	9.7	73.7	

As for the different phases of the walk, we did not find differences in step or stride length. Step length had a mean value of 1.64 ± 0.75 m and stride length had a value of 0.82 ± 0.37 m in obese subjects. Values for the controls were very similar, with a step length of 1.81 ± 0.53 m and a stride length of 0.90 ± 0.26 m.

No differences were found for the swing phase duration: 433.54 ± 104.73 ms for controls and 409.47 ± 71.57 ms for obese subjects.

The difference in cadence between the two groups (p = 0.05) was statistically significant: for the obese patients in fact we found a cadence of 93.11 ± 20.11 step/min, while for controls it was 104.18 ± 10.06 step/min. Consequently, even the difference in walking speed was significantly different (p = 0.04), measuring 66.04 ± 19.83 m/min for obese subjects and 79.32 ± 19.44 m/min for controls. It follows that stride cycle duration was significantly different (p = 0.036): 1.12 ± 0.09 s for controls and 1.18 ± 0.07 s for obese subjects.

A highly statistical difference was found in the rate of single and double limb support during the stride cycle (SLS/DLS) (p < 0.0005): 241.76 ± 37.08 for the obese subjects and 315.74 ± 50.16 for controls. Nevertheless, considering separately the duration of the single limb support (SLS) and the double limb support (DLS), only a trend toward reduction for the former and an increasing for the latter were found in the obese subjects with respect to controls, no statistically significant (Table 4).

No significant differences were found also in the duration of a single footstep: 553.22 ± 57.97 ms for controls versus 590.95 ± 76.65 ms for the obese. Neither the Pulling Acceleration nor the Swing Power and the Ground Impact showed significant differences between the two groups.

Finally, we noticed a significant reduction in the pre-swing angle in the obese subjects in comparison with the controls (p = 0.03). This value was 37.54 ± 18.02 degrees in the obese subjects against 50.14 ± 15.31 degrees in the controls.

### Energy expenditure

With regards to energy expenditure, the average (p = 0.007), total (p = 0.02), least (p < 0.0005) and maximum (p = 0.046) were significantly greater in the obese subjects than in the controls.

In the controls the average energy expenditure was 1.78 ± 0.27 kcal/min and the total was 2525.5 ± 427.63 kcal/min, which is therefore significantly lower in comparison to those found in obese patients, which were 2.30 ± 0.66 kcal/min as average energy expenditure and 3027.09 ± 731.09 kcal as total energy expenditure. As for the least energy expenditure the obese patients had values of 1.38 ± 0.18 kcal/min against 1.12 ± 0.21 kcal/min in controls. Finally, the maximum energy expenditure was 16.41 ± 4.68 kcal/min in the obese patients, and 13.48 ± 3.73 kcal/min in controls.

## Discussion

Daily physical activities have a fundamental role in all therapeutic approaches to obesity to reach a state of fitness, and adequate instruments to assess it objectively and reliably are required.

Different studies support the validity and reliability of the instrument we used (IDEEA - Minisun, Fresno, CA), to measure gait analysis parameters and energy expenditure both in healthy and unhealthy subjects, having been tested in hemiparetic subjects [18], patients with arthritic knee [22], minimally invasive total hip arthroplasty [23], high-flex total knee replacement [24], elderly [25] and children with cerebral palsy [26,27]. As the first step we considered it appropriate to compare gait analysis data, obtained in the present study for control subjects by means of the IDEEA device, with the literature. Table 5 shows data from available studies. Control data in the present study seem to compare well with the few previous findings on healthy people even if the number of subjects and the number of steps performed are greater in our study. However, we found a wide variability both in the controls and in the obese subjects, which is difficult to compare to the other studies. The only long-term monitoring study [18], reports a wide variability in the number of steps walked but unfortunately it does not present values relevant to specific measurements. The large variance might account for the fact that even if most of the parameters detected were different in the two populations, only a few were statistically significant, particularly for gait analysis. This can be considered as a statistical limitation of this pilot study which should be confirmed on a larger sample of both obese and control subjects. In this respect moreover a previous study on the reliability of the system [19] warns about the need to record a steady state ambulation outside a laboratory, which is a necessary condition for the validity of gait data.

**Table 5:** Comparison of data from literature

Parameters	Present study	Huddleston et al 2006	Saremi et al 2006	Maffiulletti et al 2008
	**15 healthy age ranged 24-69 yrs, 9173.58 ± 386.79 steps**	**5 healthy, 43.8 ± 14.5 yrs, 8441 ± 4785 steps**	**1 healthy, 56 yrs, 10 steps**	**10 healthy, 34 ± 11 yrs, 4-5 steps for each trial, 9-12 trials for individual subjects**

Stride Length (m)	1.81 ± 0.53		1.56 ± 0.05	L 1.441 ± 0.133 R 1.427 ± 0.149

Step Length (m)	0.90 ± 0.26		0.78 ± 0.03	L 0.715 ± 0.068 R 0.714 ± 0.083

Swing (msec)	433.54 ± 104.73		397 ± 14.7	

Cycle (s)	1.12 ± 0.09		0.99 ± 0.02	

Cadence (steps/min)	104.18 ± 10.06		120 ± 3.88	116.6 ± 9.8

Speed (m/min)	79.32 ± 19.44	11.7 ± 4.5	94.0 ± 2.9	1.38 ± 0.14 m/sec (82.80 m/min)

SLS (msec)	433.54 ± 104.73		397 ± 14.7	L 420.3 ± 28.7 R 419.5 ± 22.7

DLS (msec)	156.60 ± 68.63		103 ± 14.7	

SLS/DLS	315.74 ± 50.16 (%)		3.85 ± 0.52	

Pulling Accel (G)	0.62 ± 0.39		0.69 ± 0.07	

In the present study, even a trend in reduced time spent in walking and standing was present as well as an increasing in time spent sitting and a reduction in number of steps walked, these data showed large variance and were not significantly different in the two groups. Obese subjects conversely spent significant less time stepping and walked with reduced speed related mainly to reduced cadence, and reduced rate of single and double limb support (SLS/DLS). These findings are in agreement with the data previously reported on gait in obese people by means of optoelectronic systems [28,29]. The greater tendency in obese subjects to be sedentary can be explained by looking at the results of daily energy expenditure. In fact, both the average and the total values of energy expenditure were significantly greater in obese subjects, and probably this greater energetic demand makes the obese subject to leading a less active life reinforcing a vicious circle. Also this finding is in agreement with data in the literature, which show that average energy expenditure is significantly higher in obese people, resulting from an increase in the energy cost of both basal metabolism and physical activity [30-32].

The assessment of nighttime postures is worth of attention as results showed that obese patients spent more time sitting or reclining instead of lying. Previous work on the reliability of the IDEEA device in detecting position showed that the reclining posture is one of the measures most subject to error for correct identification due to anatomical differences among subjects [13,26]. The alignment of trunk sensors is, in fact, crucial to distinguish between sitting, reclining and lying down. Even if this could be the case of obese people where the anatomy of the trunk could lead to a critical detection, we feel that this could be more related to the distinction between the sitting and the reclining positions than to the lying position. In fact, direct interview to the patients revealed that most obese subjects had the habit of sleeping with more pillows because of the better respiratory dynamics achievable in that position [33]. There is evidence in fact that the raised position favors diaphragmatic work and limits the fat pressure on the hypopharynx, thus decreasing the frequency of apnea during sleep and allowing the expansion of the rib cage [34]. Nevertheless, as we did not ask subjects to keep a log of their activities, we can consider the interpretation of findings from the present work just as an hypothesis as we do not know exactly if the obese subjects stayed up late or get up early for work or other reasons and therefore spent less time in a supine position during the night hours.

The IDEEA device was very easy to assemble and calibrate. Although sometimes very obese people had some discomfort as the cabled sensors were tightened, none of the subjects included in the study reported any problem of detachment of sensors or problems with the device during the monitoring period. The main limitation of the device reported by the subjects was the impossibility to have a bath or a shower. This could be critical particularly when activity has to be monitored over periods longer than 24 hours. At the beginning of the present study our purpose was to assess patients before and after a program of cognitive-behavioral treatment for obesity. However, when we monitored subjects after 3 months program for weight loss, we realized that, whereas some subjects had a change in motor behavior, the data recorded in most subjects over 24 hours were unchanged. It is reasonable to suppose that, especially for people who work and spend most of their time in a sitting position for professional reasons, 24 hours is not long enough to observe the actual modifications of their lifestyle. Even it was previously suggested, based on the use of a pedometer, that a reasonable assessment of patient activity can be obtained in 4 consecutive days [35].

In conclusion the IDEEA device has shown to be a very useful tool, that provides objective information in the evaluation of physical activity and lifestyle in obese subjects. Its applications in the prevention of obesity and rehabilitation (reconditioning) of obese people are very promising. It can be used both as a tool for discussing objective data with patients about their habits and for measuring the effects of fitness programs in obese subjects. Further studies and optimization of wearable devices for their usability are needed for quantitative measure of physical activities over longer period of the patient's life.

## Competing interests

The authors declare that they have no competing interests.

## Authors' contributions

MGB designed the study, participated in the statistical analysis and manuscript writing; ADG and LC participated in data collection and analysis, and manuscript writing; LB participated in data collection, and statistical analysis; LDE and GT participated in the recruitment of obese subjects; NM participated in the study design and in the recruitment of obese subjects; SG participated in the study design and gave final approval to the version of the manuscript to be submitted. All the authors approved the final version of the manuscript.
